# The Feasibility and Safety of the Clip-and-Snare Method with a Prelooping Technique for Gastric Submucosal Tumors Removal: A Single-Center Experience (with Video)

**DOI:** 10.1155/2022/7957877

**Published:** 2022-08-17

**Authors:** Qi Tang, Rui-Yue Shi, Jun Yao, Li-Sheng Wang, De-Feng Li

**Affiliations:** ^1^Department of Gastroenterology, Shenzhen People's Hospital, Second Clinical Medical College, Jinan University, Guangzhou, China; ^2^First Affiliated Hospital, Southern University of Science and Technology, Shenzhen 518020, Guangdong, China

## Abstract

**Aim:**

This study compared the efficacy and safety of endoscopic submucosal dissection (ESD) combined with clip-and-snare method and a prelooping technique (CSM-PLT) with ESD alone for the treatment of gastric submucosal tumors (gSMTs).

**Methods:**

We retrospectively enrolled a matched group of 86 patients who received ESD combined with CSM-PLT or ESD alone from July 2010 to July 2020. The primary outcomes included complete resection, en bloc resection, and R0 resection.

**Results:**

Eighty-six patients with gSMTs were enrolled in ESD combined with CSM-PLT group and ESD group, respectively. There were no significant differences in gender, age, tumor size, tumor location, and tumor origin between the two groups. The complete resection, en bloc resection, and R0 resection rates were comparable between two groups (*P*=1, *P*=0.31, and *P*=0.25, respectively). There were no significant differences in terms of hospital stays, hospitalization cost, postoperative complications, and residual rate (*P*=0.42, *P*=0.74, *P*=0.65, and *P*=1, respectively) between the two groups. However, the ESD combined with CSM-PLT was associated with a shorter procedure duration and fewer intraoperative complications (*P* < 0.001 and *P*=0.024, respectively). In addition, the incidence of intraoperative bleeding in ESD combined with CSM-PLT group was significantly lower than that in ESD group (*P*=0.04).

**Conclusion:**

Both ESD combined with CSM-PLT and ESD were effective and safe modalities for the treatment of gSMTs. However, ESD combined with CSM-PLT was associated with a shorter procedure duration and fewer intraoperative complications.

## 1. Introduction

Gastric submucosal tumors (gSMTs) are commonly diagnosed without obvious symptoms, which are found incidentally during endoscopic and radiographic examinations [1]. Meanwhile, the etiology of most gSMTs is determined as a gastrointestinal stromal tumor (GIST) after resection [2, 3]. However, GIST is a potentially malignant tumor [4, 5]. Therefore, it is necessary to pay more attention to gSMTs in clinical practices.

The clinical guidelines of GIST recommend that small gSMTs less than 2 cm without malignant features (such as irregular border, necrosis, heterogeneous internal echo, and size increase) can be monitored by periodical endoscopy, endoscopic ultrasonography (EUS), and computed tomography (CT), whereas gSMTs larger than 2 cm or those with malignant features need to be removed [6–10]. Nevertheless, it may impose a tremendous emotional burden and increase the financial burden for patients during follow-up. Therefore, early intervention of gSMTs smaller than 2 cm without malignant features remains significantly necessary [11, 12].

Several laparoscopic and endoscopic modalities have been reported for the treatment of gSMTs. The laparoscopic modalities include laparoscopic intragastric surgery (LIGS), laparoscopic wedge resection (LWR), and laparoscopic subtotal gastrectomy (LSG), while endoscopic modalities include endoscopic submucosal dissection (ESD), endoscopic submucosal excavation (ESE), and endoscopic full-thickness resection (EFTR) [1, 13]. However, many studies have discovered that ESD had less invasion, shorter procedure duration, less pain, less gastric tissue resection, less bleeding, and lower cost compared with laparoscopic resection (LR) for small (less than 5 cm) and intraluminal gSMTs [14, 15].

The clip-and-snare method with a prelooping technique (CSM-PLT), as a traction method, is initially used for resection of early gastric cancer during gastric ESD, which had several advantages, such as shortened procedure duration, decreased bleeding, and obtained good visibility [16, 17]. However, few studies have evaluated the feasibility and safety of CSM-PLT for gSMTs during gastric ESD. Therefore, we retrospectively compared the feasibility and safety of ESD in combination with CSM-PLT and ESD alone for the treatment of gSMTs in a single clinical center.

## 2. Methods

### 2.1. Patients and Study Design

**Figure 1:**
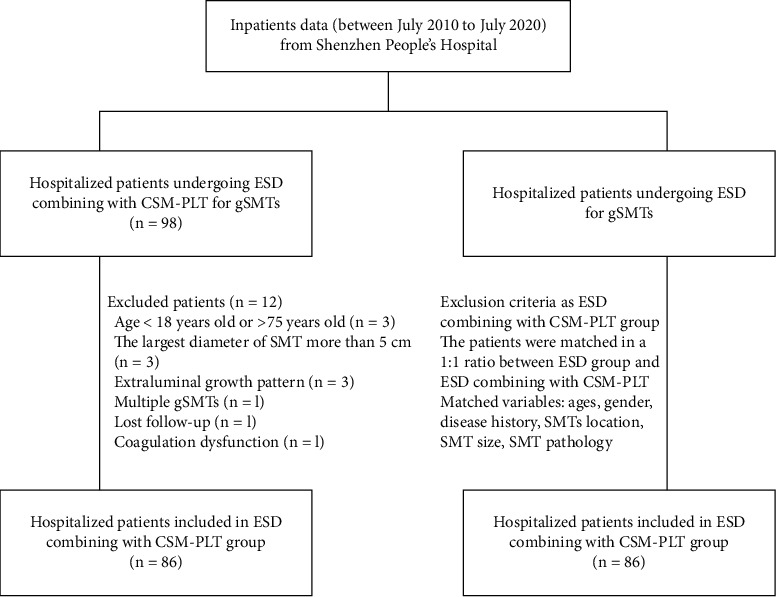
Flow chart.

A total of 98 consecutive patients have undergone ESD in combination with the CSM-PLT for gSMTs in the Second Clinical Medicine College (Shenzhen People's Hospital) of Jinan University from July 2010 to July 2020. The inclusion criteria were set as follows: (1) patients aging between 18 and 75 years old, (2) the largest diameter of the target SMT less than 5 cm, (3) intraluminal growth pattern, and (4) no high-risk features, for instance, irregular border, necrosis, heterogeneous internal echo, and size increase. The exclusion criteria were set as follows: (1) severe cardiopulmonary disease, (2) coagulation dysfunction (international normalized ratio >2.0 and platelet count <100,000/mm^3^), (3) multiple gSMTs, (4) gastrectomy, and (5) lost follow-up. According to inclusion and exclusion criteria, 86 patients were finally enrolled in the ESD in combination with CSM-PLT group (Figure 1).

For comparative assessment, 86 patients with matched baseline characteristics undergoing ESD alone for the treatment of gSMTs were obtained in the same period (Figure 1). Overall, there were 86 and 86 patients in the ESD combined with CSM-PLT group and the ESD alone group, respectively. The size and origin of the gSMTs were confirmed using EUS and computed tomography (CT). This study was approved by the Ethics Committee of Shenzhen People's Hospital. All patients provided written informed consent.

### 2.2. ESD Technique for gSMTs

ESD was performed under general anesthesia with endotracheal intubation and CO_2_ insufflation. A single-channel endoscope (GIF-260J, Olympus Co., Tokyo, Japan) was adopted, which was equipped with a disposable transparent cap on the endoscopic tip. The steps of ESD were briefly described as follows: (1) marking dots for incision lines were placed at the edge of gSMTs using KD-650Q (Endocut mode, 50 W, effect 3, ERBE, Germany). (2) Submucosal injection was performed using saline containing 0.3% indigo carmine beneath the marking dots to lift the mucosa. (3) A lateral incision was performed as deep as submucosa layer around the marking dots using Hook Knife (Endocut mode, 30 W, effect 3, ERBE, Germany). (4) Insulation-tipped knife (IT) (Endocut mode, 30 W, effect 3, ERBE, Germany) was used to dissect the gSMTs. (5) The visible vessels were coagulated. (6) The specimens were retrieved and the defects were completely closed by the clips.

### 2.3. ESD Combined with CSM-PLT for gSMTs

**Figure 2:**
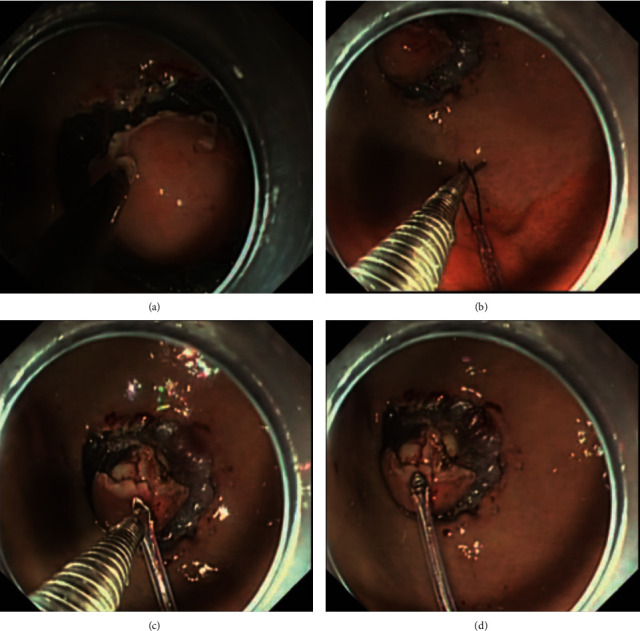
ESD combined with CSM-PLT procedure. (A) (a) Circumferential incision; (b) moving the hemoclip toward the prelooped snare; (c) releasing hemoclip and tightening the snare; (d) applying an appropriate tension to the tumor through the snare.

ESD combined with CSM-PLT was also performed under general anesthesia with endotracheal intubation and CO_2_ insufflation. Similar to ESD procedure, the ESD combined with CSM-PLT was performed by marking dots at the edge of gSMTs, submucosal injection, and a lateral incision. However, ESD combined with CSM-PLT included these main steps as follows: (1) after a circumferential incision, the endoscope was withdrawn, and the transparent cap was tightened with a snare (Micro-Tech, Nanjing, China) from outside of the endoscope (Figure 2(a)). (2) The endoscope and snare were reinserted, and a hemoclip with a reusable clip deployment device (Micro-Tech, Nanjing, China) was inserted through the endoscope channel. (3) The prelooped snare was loosened from the transparent cap and moved the hemoclip toward the prelooped snare (Figure 2(b)). (4) The hemoclip was released from the hemoclip deployment device and tightened the snare (Figure 2(c)). After these steps, the endoscopists dissected the gSMTs applying an appropriate tension to the tumor through the snare (Figure 2(d)). Finally, the specimens were retrieved, and the defects were completely closed by the clips (Video 1).

### 2.4. Perioperative Management

All patients were hospitalized and kept nil per os (NPO) for 8 h before the procedure. Antibiotics were routinely given to prevent the infection for 3 days. Moreover, all patients were intravenously administered with prophylactic proton pump inhibitors (PPIs) (Esomeprazole, 40 mg, twice daily) for 3 days, after which PPIs (Esomeprazole, 20 mg, twice daily) were orally administered for 8 weeks. If patients showed no evidence of complications for 3 days, a full fluid diet was given for the following 3 days, and normal food was gradually given in the next 2 weeks. Possible complications were monitored, such as postprocedure bleeding and perforation.

### 2.5. Pathology Evaluation

The specimens were immersed in 10% buffered formalin and then embedded with paraffin. The sectioned slices were stained with hematoxylin and eosin (H&E). Immunohistochemical staining was used to determine undefined pathological type. The risk potential of GISTs was determined following the consensus risk classification of the National Institutes of Health [18].

### 2.6. Follow-up

Surveillance endoscopy was conducted to evaluate the wound healing and monitor the residual and recurrent lesions at 3, 6, and 12 months and then repeated yearly. For patients diagnosed with GISTs, a contrast-enhanced CT was recommended every 12 months.

### 2.7. Outcomes

The primary outcomes were the rates of complete resection, en bloc resection, and R0 resection. The complete resection was defined as no residual tumor fragment at the resection site on endoscopic views. En bloc resection was defined as single piece resection. R0 resection was defined as an en bloc resection without a pathological margin.

The second outcomes included procedure duration, hospital stay, hospitalization cost, residual tumors, recurrent tumors, intraoperative complications (bleeding and perforation), and postoperative complications (bleeding and perforation). The procedure duration was defined as the time from the start of marking dots to the closure of the incisions. The hospital stay was defined as the duration from the day of operation to the discharge day. Intraoperative bleeding was defined as the requirement of endoscopic intervention during the procedure, while postoperative bleeding was defined as hematemesis, melena, or hemoglobin level of more than 2 g/dl reduction after the procedure. Intraoperative perforation was defined if the abdominal structure was visualized during the procedure, while postoperative perforation was defined as evidence of diffuse gas or fluid localized in the peritoneum.

### 2.8. Statistical Analysis

Continuous variables were expressed as mean ± standard deviation (SD) or mean (interquartile range [IQR] ranged from the first quartile (Q1) to the third quartile (Q3)) based on the distribution. Unpaired Student's *t*-test or Mann-Whitney *U* test was used to calculate continuous variables. Categorical variables were expressed as frequency (percentage). Pearson Chi-squared test or Fisher's exact test was used to calculate categorical variables. All analyses were conducted by the SPSS 23.0 software package (SPSS Company, Chicago, IL, USA). *P* values < 0.05 were set as statistical significance.

## 3. Results

### 3.1. Baseline Characteristics

From July 2010 to July 2020, 86 patients with 86 intraluminal gSMTs were treated with ESD combined with CSM-PLT in our clinical center (58.1% male, 54.3 ± 11.4 years of age). A group of 86 patients with matched baseline characteristics, such as gender, age, tumor size, tumor location, tumor original, and pathological type, received ESD alone for gSMTs. The mean tumor size in the ESD combined with CSM-PLT group was similar to that of the ESD alone group (26.5 (12.0–49.0) mm vs. 28.4 (13.0–48.0) mm, *P*=0.92). Most gSMTs were located in the gastric body and fundus in ESD combined with CSM-PLT group and ESD group (88.4% vs. 88.3%, *P*=0.97). The gSMTs originating from muscularis propria (MP) layer accounted for 95.3% and 93.0% in these two groups, respectively (*P*=0.52). The results of pathological outcomes showed that the majority of tumors were GISTs (91.9% vs. 91.9%, respectively, *P*=0.89). According to the risk classification of patients with GIST, the number of very low-, low-, medium-, and high-risk patients was comparable between two groups (*P*=0.9) (Table 1).

### 3.2. Clinical Outcomes

The rate of complete resection was not significantly different between the ESD combined with CSM-PLT group and the ESD alone group for gSMTs (100% vs. 100%, *P*=1). Compared with ESD alone, ESD combined with CSM-PLT tended to increase the rates of en bloc resection and R0 resection for the treatment of gSMTs; however, the difference was not significant (98.8% vs. 96.5%, *P*=0.31; 97.7% vs. 94.2%, *P*=0.25, respectively). There were no significant differences between these two modalities in terms of hospital stay and hospitalization cost (*P*=0.42 and *P*=0.74, respectively). However, the procedure duration was shorter in the ESD combined with CSM-PLT group than that of ESD group (*P* < 0.001) (Table 2).

### 3.3. Perioperative Complications

Two patients (2.4%) developed complications during ESD combined with CSM-PLT procedure, including one perforation (1.2%) and one bleeding (1.2%). However, three patients (3.5%) and six patients (7.0%) experienced perforation and bleeding during the ESD alone procedure. Therefore, the overall intraoperative complication was significantly lower in ESD combined with CSM-PLT group than that of ESD group (2.4% vs. 10.5%, *P*=0.0024). Although the intraoperative perforation rate was comparable between the two groups (1.2% vs. 3.5%, *P*=0.3), the intraoperative bleeding was significantly lower in ESD combined with CSM-PLT group (1.2% vs. 7.0%, *P*=0.04). Fortunately, all the complications were successfully managed endoscopically; there was no case converted to open surgery in the two groups (Table 3).

There were one delayed perforation (1.2%) and one delayed bleeding (1.2%) in the ESD combined with CSM-PLT group, while there were one patient (1.2%) and two patients (2.4%) who developed to delayed perforation and bleeding in the ESD alone group, respectively. Therefore, there was no significant difference for postoperative complication between the two groups (2.4% vs. 3.6%, *P*=0.65). Indeed, all patients with complications were recovered uneventfully after conservative treatment (Table 3).

### 3.4. Follow-up

All patients received follow-up, and the median period was 56 months (ranging from 3 to 116 months), whereas the follow-up time of ESD combined with CSM-PLT group was shorter compared with ESD alone group (65 (3–99) months vs. 73 (26–118) months, *P* < 0.001). However, no residual and recurrent lesions were detected in any of the patients during the follow-up period in both groups.

## 4. Discussion

To the best of our knowledge, we, for the first time, retrospectively compared the feasibility and safety of ESD combined with CSM-PLT and ESD alone for the treatment of gSMTs. The clinical characteristics of patients and target gSMTs were specifically matched between two groups. Our results showed that the complete resection rate was comparable between the two groups. Although the ESD combined with CSM-PLT approach tended to increase the rates of en bloc resection and R0 resection, the differences were not significant. There were no significant differences in terms of hospital stay, hospitalization cost, and postoperative complications. However, compared with ESD alone technique, ESD combined with CSM-PLT technique could not only shorten the procedure duration, but also reduce the intraoperative complications.

The results of pathological outcomes showed that the majority of tumors were GISTs. According to the risk classification of patients with GIST, most of cases were at very low risk. However, about 1.2% to 2.4% of patients are at high risk and required further intervention due to clinical guideline [19]. The proportion of heterotopic pancreas ranged from 3.4% to 4.7%, which was considered as a rare congenital abnormality during the growth and development process. Nevertheless, it has been recently reported that few unusual cases of heterotopic pancreas contributed to malignant transformations [20, 21].

In recent years, ESD, as a minimally invasive technique, is widely not only used for early gastric cancer (EGC) in patients without lymph node metastasis (LNM), but also applied to remove the gSMTs [22–24]. ESD possesses the advantages of preserving the whole stomach and maintaining the integrity of gastrointestinal tract. Therefore, it can decrease the complications and improve the quality of life [22, 23]. Meng et al. have found that although there are no significant differences regarding the rates of complete resection and complications between the ESD and LWR for gSTMs smaller than 50 mm, ESD is associated with a shorter procedure duration, a shorter hospital stay, and a lower hospital cost [14]. En bloc resection rate, procedure duration, hospital stay, and complication rate of the ESD alone group in our study were comparable to those of Meng et al. (96.5% vs. 98.5%, 84.8 ± 21.6 min vs. 89.7 ± 23.5 min, 2342 ± 512 vs. 2471 ± 573, and 14.0% vs. 11.8%, respectively). However, the ESD combined with CSM-PLT approach had a higher en bloc resection, a shorter procedure duration, and a lower complication rate compared to Meng et al. study (98.8% vs. 98.5%, 60.2 ± 17.2 min vs. 89.7 ± 23.5 min, and 4.8% vs. 11.8%, respectively) [14]. EFTR technique presents satisfactory rates of complete resection, en bloc resection, and R0 resection for gSTMs smaller than 50 mm, which are slightly higher, when compared with ESD combined with CSM-PLT technique in this study (100% vs. 100%, 100% vs. 98.8%, and 100% vs. 97.7%, respectively). However, EFTR technique is associated with dramatically higher postoperative complications (72.6% vs. 2.4%) [25]. Hoteya et al. have demonstrated that laparoscopic and endoscopic cooperative surgery (LECS) achieved the R0 resection rate of 100% with no postoperative complications for gSMTs, which is slightly superior to the ESD combined with CSM-PLT technique, whereas LECS is associated with a longer procedure duration and more invasion [26]. Zhang et al. have described a traction technique, termed pulling of the submucosal tumor with a snare combined with endoclips (PSMT-SE), to assist resection of gSMTs [27]. Meanwhile, their results showed the en bloc resection rate was 100% with no postoperative delayed bleeding or perforation, which was slightly superior to our results of the ESD combined with CSM-PLT technique (100% vs. 98.8%, 0 vs. 1.2%, and 0 vs. 1.2%, respectively) [27]. However, PSMT-SE technique was relatively complicated. Therefore, the ESD combined with CSM-PLT technique was feasible and safe for the treatment of gSMTs.

There were several strengths of the CSM-PLT technique mentioned. First, the gSMTs were lifted by applying appropriate tension during the CSM-PLT procedure, which could obtain good visibility of the operative site. A good visualization could facilitate the identification of blood vessels and MP layer, contributing to decrease of intraoperative bleeding and perforation. Second, the taut MP layer with CSN-PLT could improve the efficiency of incision, which could shorten the procedure duration. Third, CSN-PLT technique could prevent the risk of gSMTs falling into the abdominal cavity, if the patients experienced intraoperative perforation.

There were several limitations in our study. First, this was a retrospective study, and the data were obtained from one tertiary referral center. Second, it was impossible to ignore selection bias, because a case-matched comparison between the ESD combined with CSN-PLT group and ESD alone group was conducted in this study. Third, the endoscopists involved in the study were professionals in gastric ESD procedures. Therefore, we could not guarantee whether our results were generally reproducible. Fourth, the follow-up period was too short to determine the recurrence rate of gSMTs in ESD combined with CSN-PLT group.

Collectively, both ESD combined with CSN-PLT and ESD techniques were feasible and safe to remove intraluminal growth of gSMTs smaller than 5 cm. However, the ESD combined with CSN-PLT was associated with a shorter procedure duration and lower intraoperative complication. Therefore, the ESD combined with CSN-PLT might be an alternative technique for the treatment of intraluminal growth of gSMTs smaller than 5 cm. However, multicentered, prospective, randomized, and controlled trial is further required to confirm our results.

## Figures and Tables

**Table 1:** Baseline characteristics.

	ESD combined with CSM-PLT group	ESD group	*P*value
Gender (*n*, %)			
Male	50 (58.1%)	51 (59.3%)	0.88
Female	36 (41.9%)	35 (40.7%)	

Age (age)	54.3 ± 11.4	54.5 ± 11.4	0.87

Tumor size (mm)^a^	26.5 (20.7–35.0)	27.0 (20.8–35.0)	0.92

Tumor location (*n*, %)			
Gastric fundus	54 (62.8%)	55 (64.0%)	0.97
Gastric corpus	22 (25.6%)	21 (24.3%)	
Gastric antrum	7 (8.1%)	7 (8.2%)	
Gastric cardia	3 (3.5%)	4 (4.5%)	

Tumor origin (*n*, %)			
MP layer	82 (95.3%)	80 (93.0%)	0.52
Submucosal layer	4 (4.7%)	6 (7.0%)	

Histological outcomes (*n*, %)			
GIST	79 (91.9%)	79 (91.9%)	0.89
Leiomyoma	4 (4.7%)	3 (3.4%)	

Heterotopic pancreas	3 (3.4%)	4 (4.7%)	

GIST risk grading (*n*, %)			
Very low	64 (74.4%)	62 (72.1%)	0.9
Low	13 (15.1%)	15 (17.4%)	
Intermediate	7 (8.1%)	8 (9.3%)	
High	2 (2.4%)	1 (1.2%)	

*Note.* ESD: endoscopic submucosal dissection; CSM-PLT: clip-and-snare method with a prelooping technique; MP: muscularis propria; tabGIST: gastrointestinal stromal tumor; a, mean (first quartile (Q1) to the third quartile (Q3)).

**Table 2:** Clinical outcomes.

	ESD combined with CSM-PLT group	ESD group	*P*value
Complete resection (*n*, %)	86 (100%)	86 (100%)	1
En bloc resection (*n*, %)	85 (98.8%)	83 (96.5%)	0.31
R0 resection (*n*, %)	84 (97.7%)	81 (94.2%)	0.25
Procedure duration (min)	60.2 ± 17.2	84.8 ± 21.6	<0.001
Hospital stay (days)	3.8 ± 0.4	3.9 ± 0.5	0.42
Hospitalization cost ($)	2389 ± 498	2342 ± 512	0.74

*Note.* ESD: endoscopic submucosal dissection; CSM-PLT: clip-and-snare method with a prelooping technique.

**Table 3:** Perioperative complications.

	ESD combined with CSM-PLT group	ESD group	*P*value
Intraoperative complications	2 (2.4%)	9 (10.5%)	0.024
Bleeding	1 (1.2%)	6 (7.0%)	0.05
Perforation	1 (1.2%)	3 (3.5%)	0.3
Intraoperative complications	2 (2.4%)	3 (3.5%)	0.65
Bleeding	1 (1.2%)	2 (2.4%)	0.56
Perforation	1 (1.2%)	1 (1.2%)	1

*Note.* ESD: endoscopic submucosal dissection; CSM-PLT: clip-and-snare method with a prelooping technique.

## Data Availability

The data that support the findings of this study are available from the corresponding author upon reasonable request.
